# Supplementary material for: Source reconstruction without an MRI using optically pumped magnetometer-based magnetoencephalography
Source: Imaging Neurosci (Camb). 2025 May 22;3:IMAG.a.8. doi: 10.1162/IMAG.a.8 (PMC12319812; doi:10.1162/IMAG.a.8)
Supplement: Supplementary Material [file imag.a.8_supp.pdf]

Supplementary Information for:
Source Reconstruction Without an MRI using Optically Pumped Magnetometer based Magnetoencephalography

Natalie Rhodes[1], Lukas Rier[2,3], Elena Boto[2,3] Ryan Hill[2,3] and Matthew J. Brookes[2,3]*

[1]Hospital for Sick Children, 555 University Avenue, Toronto, ON, Canada

[2]Sir Peter Mansfield Imaging Centre, School of Physics and Astronomy, University of Nottingham, University Park, Nottingham, United Kingdom

[3]Cerca Magnetics Limited, Unit 7&8, Office Village, Castle Bridge Rd, Kirtley Dr, Nottingham, United Kingdom

*Corresponding author:

Prof. Matt Brookes
Sir Peter Mansfield Imaging Centre
School of Physics and Astronomy
University of Nottingham
University Park
Nottingham
NG7 2RD
UK
Tel. +44(0)1159515188
Mob. +44(0)7977552150
email. matthew.brookes@nottingham.ac.uk

Keywords: magnetoencephalography, template MRI, optically pumped magnetometers, source reconstruction, functional connectivity

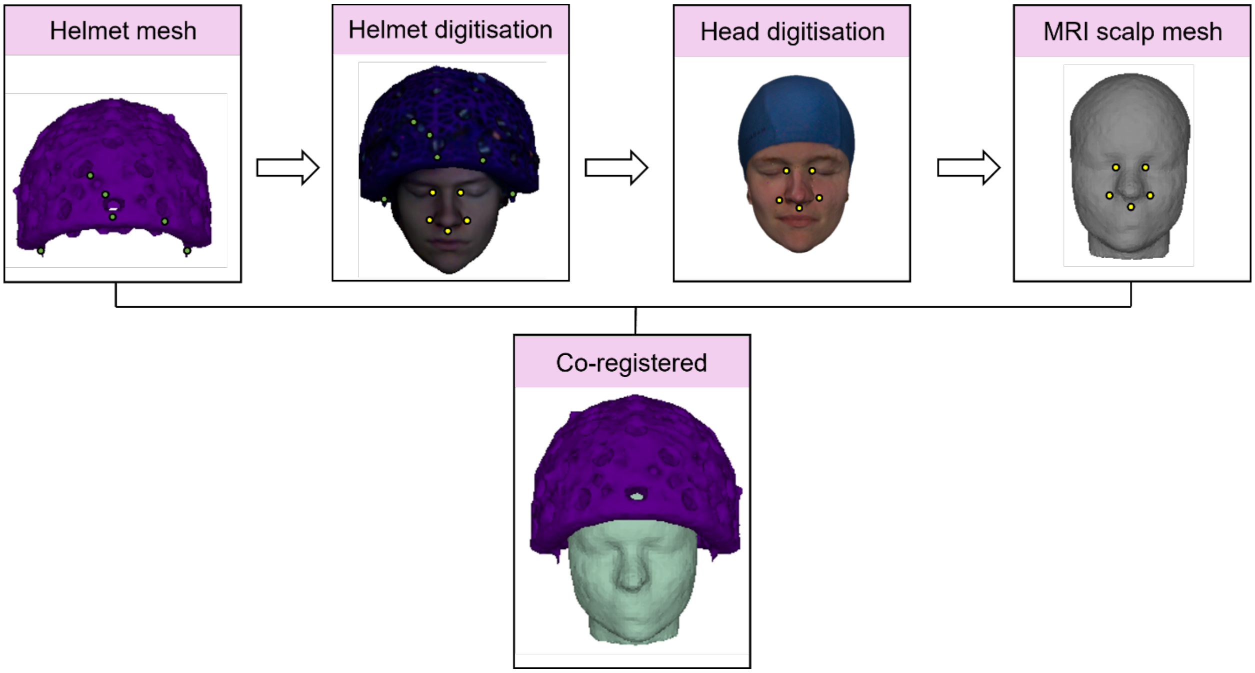

Figure S1: Optical coregistration procedure. *The digitisation of the helmet (far left) (acquired from the helmet 3D printing file) is initially aligned to the structured light (SL) scan of the helmet and face (centre left). The face (in the SL scan of the helmet and face) is then aligned to the face in the SL scan without the helmet (centre right). Finally, the face from the SL scan is aligned to the face from the MRI (far right). This results in a complete coregistration between the helmet mesh and the MRI (hence brain anatomy).*
